# Computational Modeling of Tumor Response to Vascular-Targeting Therapies—Part I: Validation

**DOI:** 10.1155/2011/830515

**Published:** 2011-03-23

**Authors:** Jana L. Gevertz

**Affiliations:** Department of Mathematics and Statistics, The College of New Jersey, 2000 Pennington Road, P.O. Box 7718, Ewing, NJ 08628-0718, USA

## Abstract

Mathematical modeling techniques have been widely employed to understand how cancer grows, and, more recently, such approaches have been used to understand how cancer can be controlled. In this manuscript, a previously validated hybrid cellular automaton model of tumor growth in a vascularized environment is used to study the antitumor activity of several vascular-targeting compounds of known efficacy. In particular, this model is used to test the antitumor activity of a clinically used angiogenesis inhibitor (both in isolation, and with a cytotoxic chemotherapeutic) and a vascular disrupting agent currently undergoing clinical trial testing. I demonstrate that the mathematical model can make predictions in agreement with preclinical/clinical data and can also be used to gain more insight into these treatment protocols. The results presented herein suggest that vascular-targeting agents, as currently administered, cannot lead to cancer eradication, although a highly efficacious agent may lead to long-term cancer control.

## 1. Introduction

Solid tumors require a functioning vasculature for the delivery of oxygen and nutrients, as well as for the removal of toxic waste products associated with cellular metabolism. A tumor can partially fill its vascular needs via the cooption (incorporation) of existing host blood vessels. However, tumor growth beyond a microscopic size and cancer cell metastasis both depend on the recruitment of new blood vessels to the tumor via a process called angiogenesis [1].

The angiogenic process is influenced by endogenous pro- and antiangiogenic molecules, as well as biophysical triggers, including metabolic and mechanical stress [1]. It is said that the angiogenic switch is “on” when the net effect of the pro- and antiangiogenic triggers is tipped in favor of angiogenesis and that the switch is “off” when the balance is tipped in the other direction [1, 2].

The growth of new blood vessels via angiogenesis invariably lags behind tumor growth. This results in a tumor vasculature that is morphologically and functionally abnormal and, hence, differs greatly from the normal adult vasculature. In particular, the angiogenic vasculature is leaky (as the vessels contain many openings), consists of many dilated vessels with varying diameter, and is highly tortuous, making blood flow through angiogenic vessels chaotic [1, 3]. Furthermore, tumor vessels tend to proliferate faster and express different proteins than the normal vasculature [4]. Taken together, these abnormal traits of the tumor vasculature allow it to be directly targeted with drugs without a significant risk of interfering with the normal vasculature [3, 4].

Vascular-targeting therapies aim to take advantage of unique features of the vascular network in tumors. These treatments fall into two general categories. The first is the *angiogenesis inhibitors* (AIs), which attempt to inhibit the tumor-initiated angiogenic process in order to prevent the formation of new blood vessels. AIs have been developed that inhibit proangiogenic molecules, bind to angiogenic receptors, inhibit the proliferation of the endothelial cells (ECs) that make up blood vessels, and upregulate/deliver antiangiogenic compounds [1, 3]. AIs are not intended to directly kill a tumor, but indirect growth inhibition and metastasis prevention are expected as the tumor cannot develop the vasculature required to maintain active growth and spread. Given the indirect mode of action of AIs, they are typically administered chronically over months and years [3].

A number of AIs are currently being tested in clinical trials as either stand-alone cancer therapies or in combination with traditional therapeutic modalities. A search at the National Cancer Institute's website (http://www.cancer.gov/clinicaltrials/search/) for “all cancers,” “treatment,” and “all countries” returns 1312 clinical trials involving antiangiogenesis compounds. A similar search on http://clinicaltrials.gov/ returns 106 clinical trials involving antiangiogenesis compounds. One AI, bevacizumab (Avastin), has been approved by the U.S. Food and Drug Administration (FDA) for use with other drugs to treat colorectal, lung, breast, brain, and kidney cancer [5]. Although bevacizumab has had transient effects in many patients and, therefore, increases progression-free survival, the long-term effects of the drug are more sobering. While many patients' exhibit an initial period of growth inhibition, tumor regrowth almost inevitably occurs after several months of treatment [5].

The second approach to targeting the vasculature involves the use of *vascular disrupting agents* (VDAs) that attempt to cause rapid and selective shutdown of tumor-associated blood vessels [3, 4]. VDAs are expected to cause cancer cell death as a result of blocking off a tumor's blood supply. VDAs achieve their selectivity for tumor-associated vessels through either ligand selectivity (i.e., selectively binding to unique angiogenic vessel receptors) or physiological selectivity [4]. Given that VDAs are expected to cause rapid shutdown of the tumor vasculature, drugs that fall into this category are designed to be used in an intermittent fashion rather than over the long-term [1].

Preclinical studies have shown that VDAs can enhance the efficacy of chemotherapy, radiation, and even antiangiogenic agents [4]. Despite the successes of preclinical trials, VDAs have not gained as much momentum as AIs in the clinical realm. A search at the National Cancer Institute's website (http://www.cancer.gov/clinicaltrials/search/) for “all cancers,” “treatment,” and “all countries” only brings up 1 VDA that is currently in clinical trials. A similar search on http://clinicaltrials.gov/ returns 13 clinical trials involving vascular disrupting agents. VDAs may be poorly represented in the clinical trial pool because while they can trigger tumor death in about 95% of a tumor mass, tumor cells tend to survive at a thin rim on the tumor periphery. This thin rim of surviving cells can eventually repopulate the mass, leading to tumor regrowth [4].

The large number of vascular-targeting compounds that are being considered for use in patients represents an exciting time for those in the field of angiogenesis research. At the same time, the discovery and development of a new drug is a time consuming and incredibly expensive undertaking. The time between preclinical testing of a compound and approval of a New Drug Application has been shown to take anywhere between 3.2 and 20 years, which an average time of about 8.5 years [6]. Further, it has been estimated that in the years 1989–2002, the final cost of developing a cancer drug averaged $1.04 billion [7]. On top of these exorbitant time and monetary costs, it is estimated that only 21.5% of drugs that complete a phase III clinical trial gain approval to be produced and marketed as a new drug [6]. Thus, given the amount of time and money it takes to develop a new drug, along with the risks of failure, pharmaceutical companies are trying to decide as early in the process as possible whether to proceed with or abandon testing a new drug.

A relatively novel method to evaluate the potential efficacy of a new compound is through mathematical/computational modeling. Experimentally validated mathematical models allow one to test the efficacy of a drug at an extremely minimal time and financial cost. In this manuscript, I have used a previously validated hybrid cellular automaton model of tumor growth to demonstrate the power *in silico* techniques have to make predictions on the antitumor activity of cancer drugs. The strengths and weaknesses of this computer-based method are discussed, and implications for the drug development process are explored.

## 2. Previous Work

The use of mathematical techniques in the drug development process is not a novel one. Pharmacokinetic (PK) and pharmacodynamic (PD) models have been utilized for decades to determine the relationship between drug dose and response. In particular, PK models study what the body does to a drug, including mechanisms of drug absorption and distribution (typically modeled through differential equations) and duration of drug effect [8]. On the other hand, PD models study the relationship between drug concentration and it physiological effects on the body. Typically, drug-receptor interactions are modeled via a system of differential equations [8]. By coupling PK and PD models, one can understand both a drug's effects on the body (at least the receptor level) and the bodies effects on a drug. PK/PD modeling has become increasingly important in the drug development process, being used in preclinical trials to support drug discovery, to interpret toxicity data [8], and to determine optimal dosing strategies [9–12].

Mathematics has been employed in other ways to study tumor response to drug administration. While a comprehensive discussion of these approaches is beyond the scope of this paper, it is worthwhile to mention a number of interesting models that have been developed to understand tumor drug response. Chemotherapy, for example, has been extensively studied using mathematical models. Some of these models focus on the predicted efficacy of a chemotherapeutic treatment regime [13–15], its dependence on the immune system [16], the transport of chemotherapeutic agents [17–19], the development of drug resistance [20–23], and, as previously mentioned, optimisation of scheduling protocols [9–12, 16, 24].

Recently, PK/PD models have been coupled with models of tumor growth in order to explore drug dynamics and the resulting impact on tumor growth rates [14, 25]. In particular, in [14], the authors developed and coupled a PK/PD model of a chemotherapeutic agent called Doxorubicin with a model of non-Hodgkin's lymphoma (a nonsolid tumor) progression. Using the mathematical model, predictions were made on the efficacy of Doxorubicin in patients with high-grade, intermediate-grade, and low-grade non-Hodgkin's lymphoma [14].

While the work done in [14] focused on nonsolid tumors, PK/PD models have been merged with models of solid tumor growth as well [25]. In particular, in [25], the authors grew *in silico* tumors via an experimentally validated multiscale model of tumor growth and angiogenesis. Once the tumors were grown, a multicompartment PK model was used to simulate chemotherapeutic agent administration. A PD model was then utilized to determine the fraction of cells whose growth was inhibited by the administration of each of the chemotherapeutic agents [25]. The novelty in this work lies in merging standard PK/PD modeling approaches with computer-based tumor growth models, allowing levels of tumor cell inhibition to be determined. In this way, the short-term effects of a drug on cancer growth can be predicted.

A strength of the aforementioned model is its ability to predict drug effects that are consistent with experimental data. However, the model does not look at the long-term effects of drug therapy. Long-term effects of cancer drug therapy have been explored elsewhere [26, 27]. In particular, in [26], the authors developed a nonlinear system of partial differential equations to study the continuous infusion of blood-borne chemotherapeutic agents that either target proliferating cells, a proangiogenic factor, or the tumor vasculature [26]. Tumor growth was compared before and after therapy, allowing the effects of different chemotherapeutic agents to be studied. In [27], a reaction-diffusion model that accounts for tumor-host interactions was utilized to test the impact of different chemotherapeutic regimes, including an AI and a cytotoxic drug that targets proliferating cancer cells. Based on the simulated spatial distribution of normoxic and hypoxic cancer cells after treatment, the capabilities of these drugs to reduce tumor mass and invasion were quantified [27]. Both of these works provide an example of how a mathematical model can be used to determine the long-term impact a drug has on tumor growth and survival.

The aforementioned models all represent progress in the direction of computer-aided drug development. To my knowledge, all models of solid tumor growth that have been developed for this purpose considered tumors that grow in avascular environments. On the other hand, a number of malignancies, including brain cancer, grow in vascular environments. Given the important role the tumor microenvironment plays in treatment response, it is important to expand the modeling work to include tumors that grow in well-vascularized environments.

## 3. Mathematical Model

### 3.1. Hybrid CA Model of Vascular Tumor Growth

The mathematical model I will utilize to test tumor response to an array of vascular-targeting therapies was developed to simulate the growth of a particular type of brain cancer called glioblastoma multiforme (GBM). GBM is the most aggressive of the gliomas, a collection of tumors arising from the glial cells or their precursors in the central nervous system [28]. Despite advances made in cancer treatment, the median survival time for a patient diagnosed with GBM is currently between 12 and 15 months [29]. While this prognosis is grim, this median represents a significant improvement over the 2001 median survival time, which was only eight months [30].

Despite some measurable improvements in GBM survival times, the disease is almost uniformly fatal. In order to understand what is unique about GBM that enables it to successfully evade treatment, Kansal et al. developed a cellular automaton (CA) model of GBM growth. In this CA algorithm, it was shown that three-dimensional tumor growth and composition can be realistically predicted by a simple set of automaton rules and a set of four microscopic parameters that account for the nutritional needs of the tumor, cell-doubling time, and an imposed spherical symmetry term [31].

The success of this CA model is in part related to its simplicity, and one of the simplifying assumptions is that the vasculature is implicitly present and evolves as the tumor grows. In order to incorporate a higher level of biological realism into the original CA algorithm, a two-dimensional hybrid cellular automaton model was developed to explore the feedback that occurs between a growing tumor and the evolving host blood supply [32]. Again, it is important to note here that the model is built under the assumption that a tumor is growing in a *well-vascularized* environment, like the brain.

For tumors growing in a vascularized environment, the cooption-regression-growth experimental model of tumor vasculature evolution has been proposed [33]. In this model, as a tumorous mass grows, the cancer cells coopt the mature blood vessels of the surrounding tissue. Given that the coopted vessels were part of the regular vasculature that provides healthy tissue with oxygen and nutrients, these vessels are generally mature and stable. While many proteins contribute to this phenotype of the normal vasculature, angiopoietin-1 (Ang-1) is constitutively expressed by normal blood vessels and plays an important role in vessel maturity and stabilization [34].

As a tumor mass grows and coopts the blood vessels of healthy tissue, the naturally-occurring antagonist of Ang-1, angiopoietin-2 (Ang-2) is thought to be upregulated by both the tumor and the surrounding microenvironment [33]. The fact that Ang-2 is an antagonist to Ang-1 means the two proteins compete for binding to a common receptor, in this case Tie-2. Given that Ang-2 limits the action of Ang-1, Ang-2 is responsible for the *destabilization* of the vasculature [33].

The fate of an unstable blood vessel depends on the presence of a third protein, vascular endothelial growth factor (VEGF). VEGF functions as a potent permeability-inducing agent, an EC chemotactic agent, an EC proliferative factor, and an anti-apoptotic signal for ECs [35]. In the presence of VEGF, unstable vessels survive in spite of their instability. However, in the absence of VEGF, the upregulation of Ang-2 relative to Ang-1 destabilizes the coopted vessels within the tumor and marks them for regression [33]. Vessel regression in the absence of vessel growth leads to the formation of hypoxic regions in the tumor mass. Hypoxia induces the production of VEGF, stimulating the growth of new blood vessels.

The algorithm utilized in this manuscript is a slightly modified version of the algorithm presented in [32]. Besides these small modifications, which are detailed below, the algorithm has also been adapted to account for the administration of a drug at predefined time intervals. The skeleton framework of the algorithm is summarized in Algorithm 1. In Section 3.2, I will go into more detail about the treatment protocol. 

**Figure 1:**
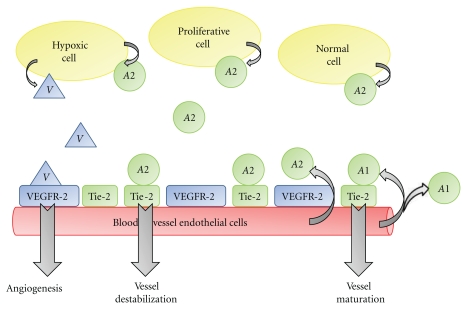
Schematic representation of system of PDEs given in (1), showing the interactions between growth factors, receptors, ligand-receptor complexes, and cell types. The ligand VEGF is denoted by *V*, Ang-1 by *A*1, and Ang-2 by *A*2. Curved arrows indicate the cell type that produced the referenced protein (e.g., hypoxic cells produce VEGF and Ang-2, whereas ECs produce Ang-1 and Ang-2), and straight arrows indicate the physiological response to ligand-receptor binding (e.g., VEGF binding to VEGFR-2 induces angiogenesis). Notice how VEGF and Ang-2 diffuse in the extracellular space, whereas Ang-1 only acts locally.

 (i) Automaton cell generation. A Voronoi tessellation of random points generated using the nonequilibrium procedure of random sequential addition of hard disks determines the underlying lattice for the algorithm [31, 32]. Each automaton cell created via this procedure represents a cluster of biological cells. Assuming the tumor under consideration is GBM (in which glial cells have an average diameter of 40 *μ*m [36]), each automaton cell is chosen to represent a cluster of seven glial cells. This number is small enough to give an average automaton cell diameter less than the characteristic diffusion length of oxygen, but large enough to keep the run-time of the algorithm manageable [32].

(ii) Healthy microvascular network. The blood vessel network which supplies the cells in the tissue region of interest with oxygen and nutrients must be generated. This is done using a modification of the Krogh cylinder model, a model of the capillary network which assumes that capillaries are straight, parallel and uniformly spaced [37]. The random analog proposed in [32] takes the idea of using parallel line segments and randomizes it, subject to a set of biologically inspired constraints. In particular, linear blood vessels are sequentially attempted to be placed within the tissue region of interest. A vessel can only be added to the system, however, if it is not too close to a parallel vessel, if it does not cause too many vessels to intersect at one site, and if it vascularizes at least one unvascularized cell [32].

(iii) Initialize tumor. Designate the automaton cell in the center of the tissue space as a proliferative cancer cell. This is equivalent to taking the nonmalignant cell in the center of the tissue and endowing it with a malignant phenotype.

(iv) Tumor growth algorithm. Time is then discretized into units that represent one real day. At each time step:

(1) *Solve PDEs*. The following previously-developed system of partial differential equations [32] is numerically solved one day forward in time
  (1) $$\begin{array}{cc} \frac{\partial v}{\partial t} & =D_{v}\Delta v+b_{v}h_{i}\left(h-\frac{v^{2}}{K_{v}}\right)-k_{0}vr_{v0}+k_{-0}r_{v}-\mu_{v}v,\\ \frac{\partial a_{1}}{\partial t} & =b_{a1}e_{i}\left(p_{i}+h_{i}+n_{i}\right)\left(e_{0}-\frac{a_{1}^{2}}{K_{a}}\right)\\ & \;-k_{1}a_{1}r_{a0}+k_{-1}r_{a1}-\mu_{a1}a_{1},\\ \frac{\partial a_{2}}{\partial t} & =D_{a2}\Delta a_{2}+b_{a2}e_{i}\left(p_{i}+h_{i}+n_{i}\right)\left(e_{0}-\frac{a_{2}^{2}}{K_{a}}\right)\\ & \;+{\bar{b}}_{a2}h_{i}\left(h-\frac{a_{2}^{2}}{K_{a}}\right)-k_{2}a_{2}r_{a0}+k_{-2}r_{a2}-\mu_{a2}a_{2},\\ \frac{\partial r_{v0}}{\partial t} & =-k_{0}vr_{v0}+k_{-0}r_{v},\\ \frac{\partial r_{a0}}{\partial t} & =-k_{1}a_{1}r_{a0}+k_{-1}r_{a1}-k_{2}a_{2}r_{a0}+k_{-2}r_{a2},\\ \frac{\partial r_{v}}{\partial t} & =k_{0}vr_{v0}-k_{-0}r_{v},\\ \frac{\partial r_{a1}}{\partial t} & =k_{1}a_{1}r_{a0}-k_{-1}r_{a1},\\ \frac{\partial r_{a2}}{\partial t} & \begin{array}{c} =k_{2}a_{2}r_{a0}-k_{-2}r_{a2}. \end{array} \end{array}$$
  A schematic of the interactions between the growth factors, receptors, ligands, ligand-receptor complexes, and cell types represented in (1) is provided in Figure 1. In these equations, *v* represents the concentration of VEGF, *r*_*v*0_ is the concentration the unbound VEGFR-2, and *r*_*v*_ is the VEGFR-2 receptor bound by VEGF. Further, the concentration of Ang-1 is given by *a*_1_, of Ang-2 is given by *a*_2_, of unoccupied Tie-2 is given by *r*_*a*0_, of Tie-2 bound by Ang-1 is given by *r*_*a*1_, and of Tie-2 bound by Ang-2 is given by *r*_*a*2_.
For the three ligands (VEGF, Ang-1, and Ang-2) each equation indicates that the protein is produced by the appropriate cell type (with a carrying capacity term [38]), and that there is a linear decay term. Both VEGF and Ang-2 diffuse, whereas Ang-1 does not. This is because Ang-1 is produced by ECs, and then acts in a paracrine manner upon these ECs [38]. Further, the source term of each protein depends on the cell types that produce the protein. VEGF is produced by hypoxic cells [38] (*h*_*i*_, where *h* stands for hypoxia and the subscript *i* denotes that this is an indicator function), Ang-2 is produced by ECs associated with malignant tissue (this includes ECs associated with proliferative cells *p*, necrotic cells *n*, and hypoxic cells) and is also produced by hypoxic cells [38], and Ang-1 is produced by ECs associated with malignant tissue.
For each receptor (VEGFR-2 and Tie-2), the equation represents the association and dissociation of the ligand-receptor complex. A complete list of variable and parameter definitions is given in Table 1. Details on the stable finite difference scheme used to solve the differential equations are found in [32].
(2) *Vessel Evolution*. Check whether each vessel meets the requirements for regression or growth. Vessels with a concentration of bound Ang-2 six times greater than that of bound Ang-1 regress [34], provided that the concentration of bound VEGF is below its critical value. Vessel tips with a sufficient amount of bound VEGF sprout along the VEGF gradient.
(3) *Nonmalignant Cells*. A healthy cell undergoes apoptosis if vessel regression causes its oxygen concentration to drop below a critical threshold. To simulate this, I use the fact that the characteristic diffusion length of nutrients in tissue is 250 *μ*m [19, 39], and I assume that oxygen can only reach cells within this critical distance from a blood vessel. Therefore, I suppose that if the distance of a healthy cell from a blood vessel exceeds the distance *l*_prolif_ = 250 *μ*m, then the oxygen level at that cell is insufficient and the cell undergoes apoptosis. Further, nonmalignant cells do not divide in the model, which is a reasonable assumption for GBM [40].

(4) *Necrotic Cells*. Tumorous necrotic cells are inert.

(5) *Hypoxic Cells*. A hypoxic cell turns proliferative if it is within a distance of *l*_prolif_ = 250 *μ*m from a blood vessel. This is equivalent to saying its oxygen level exceeds a specified threshold [32]. Similarly, a hypoxic cell turns necrotic if the oxygen level drops below a specified threshold. This is implemented by converting any hypoxic cell that is further than a distance of *l*_hyp_ = 1500 *μ*m from a vessel into a necrotic cell [32].

(6) *Proliferative Cells*.
  - A proliferative cell turns hypoxic if its oxygen level drops below a specified threshold, that is, if it is further than a distance of *l*_prolif_ = 250 *μ*m from a blood vessel.
  - If the oxygen level at a proliferative cell is sufficiently high, the cell *may* attempt to divide into the space of a viable nonmalignant cell. To determine the position of the daughter cell, an intercellular mechanical stress growth process is assumed [31]. In this process, the new proliferative cell is placed in the position of the dividing cell's nearest neighbor. If this cell is occupied (meaning if a cancer cell is already located at this nearest neighbor site), the tumor cells are successively pushed outward, eventually resulting in the presence of one new proliferative automaton cell at the tumor periphery.
  - The probability that a proliferative cell divides, *p*_div _, is influenced by the location of the dividing cell from the tumor center (*r*), reflecting the effects of mechanical confinement pressure imposed by the skull. In particular, assuming a maximum tumor extent of *R*_max_ (taken to be 10 mm in the model) and assuming that mechanical confinement pressure inhibits tumor growth, gives the following equation for *p*_div _:
    (2) $$\begin{array}{c} p_{\mathrm{div}\;}=p_{0}\left(1-\frac{r}{R_{\max\;}}\right). \end{array}$$
    The base probability of division, *p*_0_, depends on the distance of the cell to the nearest blood vessel, *d*_vessel_. The average value of *p*_0_ was fixed to be 0.192 (corresponding to a cell doubling time of approximately four days), with *p*_0_ taking on a minimum value *p*_min_ of 0.1 and a maximum value *p*_max_ of 0.284 [41]. This means that a proliferative cell in the model can have a cell doubling time anywhere in the range of three to seven days. The formula used to determine *p*_0_ is
    (3) $$\begin{array}{c} p_{0}=\frac{p_{\min\;}-p_{\max\;}}{l_{\text{prolif}}}d_{\text{vessel}}+p_{\max\;}, \end{array}$$
    where *d*_vessel_ ≤ 250 since only well-oxygenated cells can divide. Under this condition, *p*_0_ > 0.

(v) Apply treatment (if applicable on a particular day).

Importantly, the algorithm described above has been shown to be predictive (1) when a tumor can initiate angiogenesis and (2) when angiogenesis cannot be initiated [32]. In particular, it has been shown that, over an order of magnitude in tumor radius, this algorithm can successfully predict tumor size and the percent of proliferative cells found in the tumor mass [31, 32]. Further, when a tumor cannot initiate angiogenesis, the algorithm successfully predicts that the tumor cannot grow beyond a microscopic size of 1-2 mm in diameter [32, 42].

### 3.2. Treatment Protocol

In the current manuscript, the goal is to validate that the hybrid CA model can accurately predict the efficacy of a number of cancer drugs. Once the model has been shown to work on drugs of known efficacy, the model can then be used to predict the efficacy of novel therapeutic compounds.

The predictive abilities of the model will be tested using both an angiogenesis inhibitor and a vascular disrupting agent. The simulated AI will be based on the previously-discussed FDA-approved AI, bevacizumab. Bevacizumab is a monoclonal antibody that binds to and inhibits VEGF [43]. It has been demonstrated that bevacizumab encourages tumor shrinkage, increases progression-free survival times, and improves overall survival in patients with recurrent GBM [46, 47]. Given the success of phase II clinical trials, bevacizumab has been approved (through an accelerated process) for the treatment of GBM [48].

The second class of vascular-targeting agents I will explore the efficacy of are the VDAs. VDAs differ from AIs in that they selectively target the tumor vasculature for destruction. The simulated VDA will be based on a compound currently being tested in clinical trials, combretastatin A4 phosphate (CA4P). CA4P is a prodrug that is rapidly dephosphorylated to the active product tubulin inhibitor CA4 [45]. In experimental tumors, CA4P administration results in rapid and selective tumor-vascular damage. Within one hour of treatment time, blood flow through the tumor is reduced to levels of less than 5% the starting value, leading to the formation of large necrotic regions within the tumor [4].

In order to simulate drug delivery, a dosing strategy must be chosen (see Table 2). As detailed below, the dosing strategy for the simulated AI and VDA will differ significantly in order to accurately represent the use of these drugs in the clinic. For both the simulated AI and VDA, I assume the drug uniformly distributes itself throughout the vasculature. It is then assumed that, just as with oxygen, any region of tissue within a fixed distance of a blood vessel has equal access to the drug, but tissue regions further than this critical distance receive insufficient levels of the drug. Implicit in this assumption is that the drug and oxygen have the same diffusion length, which may or may not be the case. Further, the assumption of a uniform spatial distribution of the drug through the vasculature is not physiologically accurate. It is well known that variations in the vascular network and brain microstructure can impact drug delivery [1, 3, 49]. Despite this, the mathematical model proposed herein can still be used to differentiate between plausible and implausible therapies. To elaborate, a treatment that is not successful under this simplified condition has little to no hope of working under less ideal circumstances, where the drug is heterogeneously distributed throughout the tumor. Treatments that appear to thwart tumor growth in this simplified scenario are therefore *plausible* therapies that may work in a heterogeneous environment. If a plausible therapy is indeed identified, the spatial distribution of the drug can be modeled more accurately.

The following treatment scenarios will be analyzed in this manuscript.

- *AI in isolation*. I will administer a bevacizumab-like AI by inhibiting the production of VEGF (the *b*_*v*_ parameter in the model) by a factor of *T*_1_. (Note, I choose the notation *T*_*i*_ to stand for Treatment parameter *i*. This notation should not be taken to mean that all treatment parameters have the same meaning/units.) I start with the assumption that the treatment parameter *T*_1_ takes on the value of 100, and I perform a sensitivity analysis of this parameter. The AI in the simulation will be administered once every two weeks [43]. This time interval has been chosen because the half-life of bevacizumab is approximately 20 days, with effective concentrations being found in the brain two to three weeks after administration [43]. Thus, as a first pass, I assume the AI is always present at therapeutic concentrations in the brain, as administration every two weeks should ensure this occurs. Importantly, the mode of action of the AI in the model could be simulated in a number of other ways. For instance, the AI could sequester unbound VEGF and therefore limit the amount of VEGF available to bind to VEGFR-2. This is equivalent to decreasing *k*_0_, the the association rate of VEGF and VEGFR-2 in the model. Another way the AI could be modeled is by reducing the response of ECs to the presence of VEGF. This is equivalent to increasing the VEGF threshold parameter *rv*_crit_ (see [32]).

(ii) *AI with cytotoxic chemotherapy*. For those tumor types that it has been approved for, bevacizumab is typically administered in combination with a cytotoxic chemotherapeutic that targets actively dividing cells. Given that the mathematical model in this manuscript was developed to study GBM, the cytotoxic agent simulated will be the standard one used for GBM care, temozolomide [50]. It has been shown that a continuous administration schedule for temozolomide can be sustained for six to seven weeks [50]. Therefore, the cytotoxic chemotherapeutic in the model will be administered daily for a six week period of time, at which point the therapy will be discontinued for safety reasons. Given that the half-life of temozolomide is 1.8 hours [44], it can be assumed that therapeutic concentrations of the drug are maintained in the brain each day the cytotoxic agent is administered. In the model, the cytotoxic agent has a 34% chance (treatment parameter *T*_2_ = 0.34) of destroying an actively dividing cell each day a therapeutic level is maintained in the brain. This number was determined by considering the net cell kill of temozolomide over five days of treatment in mice harboring high-grade gliomas (measured to be 0.4 log units [51]) and the daily growth rate of GBM (using the fact that glioma cell doubling time is approximately four days [31]). In calculating this percent, I assumed the tumor is growing at an exponential rate and that a set percent of cancer cells are killed with each daily dose of chemotherapy (see the appendix for details). The latter assumption is referred to as the cell kill theory or fractional kill hypothesis [52]. A sensitivity analysis will be performed on *T*_2_, the cytotoxicity parameter value. Further, for these simulations, the AI will be administered as described previously.

(iii) *VDA in isolation*. I will administer a CA4P-like VDA by assuming that during each period of drug administration, there is a 60% chance (treatment parameter *T*_3_ = 0.6) the VDA destroys an angiogenic vessel. This is simulating the fact that VDAs selectively target blood vessels that grow via angiogenesis, and not the coopted vessels. Although this parameter value has been arbitrarily assigned, a sensitivity analysis will be performed for 0.1 ≤ *T*_3_ ≤ 0.9. The maximum value of *T*_3_ = 0.9 is based on the fact that, for *in vivo* studies of human breast cancer models in which there was systemic drug delivery, functional vascular volume was reduced by 93% at six hours following drug administration [53]. Therefore, the value of 93% must be a strict upper bound on the efficacy of a VDA for clinical tumors growing in vascular environments, as drug efficacy in clinical tumors is limited by the tumor microenvironment. In Phase I clinical trials, CA4P was administered intravenously once every three weeks [45]. The half-life of the prodrug CA4P was 0.47 hours, and the half-life of the active CA4 was 4.2 hours [45]. Therefore, unlike with the AI, the simulated drug cannot be assumed to always be present at therapeutic concentrations in the brain. Further, preclinical studies have demonstrated that CA4P achieves maximal vascular shutdown at four to six hours after exposure and sustained activity for up to 24 hours [45]. Thus, as a first pass at modeling VDA administration, the simulated drug will be administered once every three weeks, and the drug will only exert its effects on the vasculature the day that it is administered.

For each of the treatments (whose parameters are summarized in Table 1 and whose dosing schedules are summarized in Table 2), 10 simulations will be run and the average tumor response to the drug will be reported. Each treatment is applied once the tumor reaches the critical size of 4 mm in radius. For each of the therapeutic regimens, a sensitivity analysis of the treatment parameter(s) will also be performed. All simulations were run on a computational cluster consisting of 26 dual Opteron 248 nodes with 2 GB RAM and a processor speed of 2.2 GHz. The simulations described herein (of at least one year of physical tumor growth) took anywhere from 45 minutes to 80 minutes to complete. In the visualizations of the tumor that will be shown, the following convention is utilized: viable nonmalignant cells are labelled white, nonmalignant cells that have undergone apoptosis are green, necrotic tumor cells are black, nonproliferative/hypoxic tumor cells are yellow, and proliferative tumor cells are blue. Further, in the visualisation of the vasculature, the following convention is used: vessels that are originally part of the healthy tissue vascular network are labelled red, and vessels that grow via angiogenesis are labelled purple.

## 4. Results and Discussion

### 4.1. AI in Isolation

**Figure 2:**
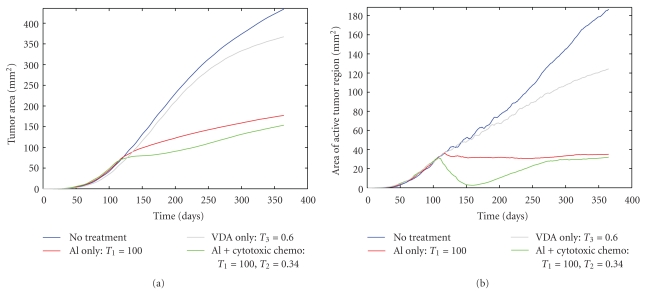
(a) Average area of tumor region and (b) average area of active tumor region, both compared for four different scenarios: no therapy is administered, AI administration only, AI with cytotoxic chemotherapy and VDA administration only.

**Figure 3:**
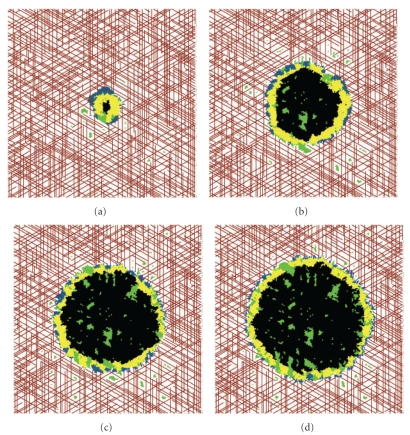
Snapshots of a tumor treated with an AI only. (a) Tumor after two months of growth, before treatment is applied. (b) Tumor after four months of growth, two weeks after treatment is first administered. (c) Tumor after eight months of growth, 19 weeks after treatment is first administered. (d) Tumor after one year of growth, 37 weeks after treatment is first administered.

As described in the treatment protocol, the first therapy tested is the administration of an AI such as bevacizumab. In Figure 2, I show the antitumor activity of the AI, as compared to the case where no treatment is utilized. In particular, I show the tumor area as a function of time (Figure 2(a)) and the active tumor area (meaning, the area of the proliferative and hypoxic regions of the tumor—Figure 2(b)), both averaged over 10 simulations. By comparing the growth of the entire tumor mass with AI treatment and without (Figure 2(a)), a clear decrease in the rate of tumor expansion is observed. Further, when looking only at the area of the active tumor region (Figure 2(b)), it is observed that this region essentially stabilizes, with no measurable growth or shrinkage after drug administration. Therefore, despite the extreme decrease in active tumor growth rate, the active tumor region is not eliminated by AI treatment. This observation is confirmed by looking at simulated images of an AI-treated tumor (Figure 3). In this figure, it can be seen that AI administration leaves a number of hypoxic and proliferative cells remaining at the tumor periphery, and this active region leads to slow growth of the tumor mass observed in Figure 2(a). The survival of active tumor cells and the resulting slow growth is largely a consequence of the fact that the tumor grows in a well-vascularized environment, highlighting the important role the microenvironment plays in treatment response.

 The results shown in Figures 2 and 3 were all obtained by decreasing the production rate of VEGF to simulate AI action. Two other modes of AI action, decreasing the association rate of VEGF/VEGFR-2 and reducing the sensitivity of ECs to the presence of VEGF, were also considered. The growth curves that resulted from the three modes of action were very similar (data not shown). Decreasing the production rate of VEGF did prove to be slightly more efficacious than the other implementation strategies, and, hence, why this mode of action was used throughout the manuscript.

**Figure 4:**
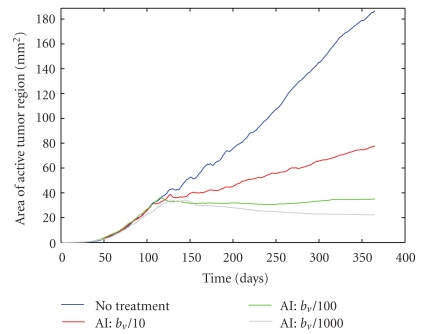
Sensitivity analysis of the AI treatment parameter. The treatment parameter was tested over two orders of magnitude, and the average area of the active tumor region predicted by the algorithm is shown for each parameter value.

A sensitivity analysis of the treatment parameter reveals that a bevacizumab-like drug will lead to a significant clinical response at all levels of inhibition tested, although the larger the efficacy of the AI, the more measurable the antitumor activity (Figure 4). In fact, when the most efficacious AI is administered (*T*_1_ = 1000), only approximately 1% of the active cell population remaining after eight months of AI treatment are proliferative cancer cells. In other words, this highly efficacious AI has almost entirely reduced the tumor to a mass of hypoxic cells; through this mass does maintain slow growth due to the surviving population of proliferative cells. Whether this level of drug efficacy is clinically achievable is not clear.

The effects of AI administration predicted by the model, particularly for *T*_1_ = 10 and 100, are in good agreement with clinical data [5]. Clinical observations revealed that when a patient is treated with bevacizumab, initial transitory improvements are observed, just as the simulations show a significant decrease in the rate of tumor expansion (Figure 2). However, in spite of these transitory improvements, clinical tumors are observed to continue growing, just as is seen in the simulations (Figures 2 and 3). Several mechanisms have been implicated in the apparent acquired resistance to bevacizumab. One hypothesis is that angiogenic tumors can evolve in the presence of an AI. For instance, evidence suggests that if one part of the angiogenic cascade is blocked, it can compensate by activating alternative proangiogenic pathways [5]. Other compensatory mechanisms tumors have developed to bypass the angiogenic blockade imposed by an AI include recruiting proangiogenic cells from bone marrow and activation/enhancement of invasion [5], which gives cancer cells access the normal tissue vasculature and makes them less dependent on the angiogenic blood supply.

While it is plausible that any or all of these mechanisms may lead to compensatory angiogenesis in clinical tumors, the simulations suggest that a tumor growing in a vascular environment can continue to grow during AI administration, even without the previously-mentioned compensatory angiogenic mechanisms. Given that the current implementation of the model does not incorporate alternative pathways that trigger angiogenesis and does not include the bone marrow or invasion, this may explain why the model predicts slow continuing growth instead of the more accelerated growth observed in patients. However, it still reveals a very important characteristic of AI treatment: innate limitations in AI treatment, and not acquired resistance, may be a limiting factor in using AIs as a single front line cancer treatment. While researchers are currently working to develop angiogenic drug cocktails that target different angiogenic pathways (with the aim of preventing regrowth), such a drug cocktail may still suffer from the same downfall as a single AI – slow growth at the tumor periphery can persist when using an AI in isolation, in particular for tumors growing in a well-vascularized environment.

### 4.2. AI with Cytotoxic Chemotherapy

My analysis of administering an AI in isolation shows that it can significantly hinder tumor growth, but it cannot eliminate the active tumor region and, therefore, slow tumor growth persists despite drug administration. However, it is not common that an AI (or a combination of AIs) would be the only form of therapy used to treat cancer. Traditional forms of therapy, including cytotoxic chemotherapy and radiation, are still used for essentially all cancer patients, provided that a patient can physically withstand the treatment. Although it may seem counterintuitive to administer an AI and a cytotoxic chemotherapeutic simultaneously, as interfering with the tumor vasculature would seem to interfere with the delivery of the chemotherapeutic, this is common clinical practice. The wisdom of this approach is something that I will explore further in future work. Currently, I will focus on analyzing the efficacy of administering an AI like bevacizumab in combination with a standard cytotoxic chemotherapeutic like temozolomide. In Figure 2, I show the average antitumor activity of a treatment protocol that involves administering an AI every two weeks for a nine month period of time, coupled with the daily administration of a cytotoxic agent for a six week period of time.

As Figure 2 shows, the combination of an AI and a cytotoxic agent has more antitumor activity than the administration of an AI in isolation. Overall tumor growth occurs at a slower rate, and the active tumor region actually shrinks while the cytotoxic therapy is being applied. In order to understand why the cytotoxic agent has this additive effect when administered with an AI, it is useful to refer to the snapshots of the simulated tumor shown in Figure 3. When an AI is applied in isolation, a small number of proliferative cancer cells survive at the tumor rim. These proliferative cells mainly obtain their oxygen and nutrients from the vasculature of the healthy tissue that surrounds the tumor mass, although some angiogenic vessels also supply the tumor. The addition of a cytotoxic agent that targets the actively dividing cells leads to the death of the proliferative cells found at the tumor periphery, and this is what causes the decrease in the active tumor area observed in Figure 2(b). The decrease in active tumor area is only maintained during the cytotoxic agent treatment window.

**Figure 5:**
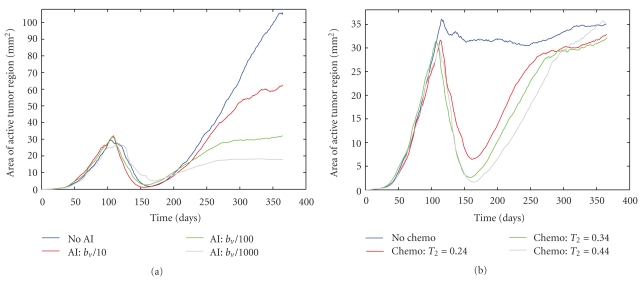
(a) Sensitivity analysis of the AI parameter when the cytotoxic chemotherapy parameter is fixed at *T*_2_ = 0.34. (b) Sensitivity analysis of the cytotoxicity parameter when the AI parameter is fixed at *T*_1_ = 100.

A sensitivity analysis of both the AI parameter (*T*_1_) and the cytotoxicity parameter (*T*_2_) has also been performed. I first focus on the case where I fix the cytotoxicity parameter at *T*_2_ = 0.34 and vary the AI parameter (Figure 5(a)). Simulations reveal that during the cytotoxic chemotherapy treatment window, the AI parameter *T*_1_ does not have a significant impact on the rate of decline of the active tumor area. In other words, when coupled with an effective cytotoxic agent, the action of the cytotoxic agent drives tumor response more so than the action of the AI. In fact, the rate of decrease in active tumor area during cytotoxic drug administration is comparable whether no AI is given, or whether the AI is administered at very high concentrations. Therefore, it seems that there is little clinical benefit for administering an AI at the same time a cytotoxic agent is being administered. However, the AI still adds significant clinical benefit to standard a chemotherapy regimen, as it does drastically control the rate of tumor expansion after removal of the cytotoxic agent (Figure 5(a)). Finally, it should be noted that if the tumor mass contains a chemo-resistant population (i.e., a population of cells that do not respond to the cytotoxic agent), simulations reveal that there is a small clinical benefit to administering an AI during the cytotoxic agent treatment window (data not shown).

Shifting to the case where the AI parameter is fixed at *T*_1_ = 100, and the cytotoxicity parameter *T*_2_ varies (Figure 5(b)) shows that *T*_2_ controls the rate of decline of the active tumor area when the treatment is first applied, but has no impact on the rate of active area regrowth upon removal of the cytotoxic agent. In fact, after 7 months off of the cytotoxic chemotherapy, the active tumor area almost catches up to the active tumor area when no cytotoxic agent was used. This suggests that a cytotoxic agent can temporarily shrink a tumor mass, therefore alleviating symptoms and possibly improving quality of life. However, this combined treatment strategy is no more effective in the long-term than applying an AI in isolation.

**Figure 6:**
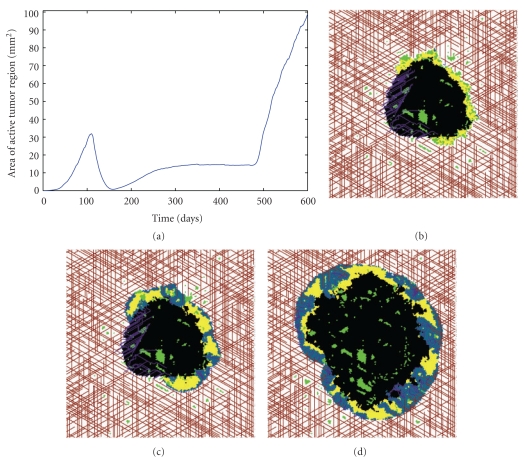
Failure of combination treatment (AI with cytotoxic agent) to limit tumor growth when the cytotoxic agent is removed after six weeks and AI is removed after one year. (a) Area of active tumor region as a function of time. (b) Snapshot of growing tumor after 10 months of treatment with AI. (c) Snapshot of tumor three weeks after ending treatment with AI. (d) Snapshot of tumor four months after ending treatment with AI.

 If we focus our attention on the most efficacious treatment parameters, *T*_1_ = 1000 and *T*_2_ = 0.44, we observe that disease stabilization can be achieved if the AI continues to be administered once every two weeks following the removal of the chemotherapeutic (see Figure 6(a), time ≤ 475 days). However, there have been reports of serious and life-threatening bleeding in patients treated with bevacizumab [54], so this drug cannot be administered indefinitely. Therefore, I ran further simulations to determine tumor response after AI removal. To simulate this scenario, the AI is removed after one year of treatment. As can be seen in Figure 6, cessation of AI treatment restimulates tumor growth, as the energy supply of dormant cells is replenished once angiogenesis is reinitiated. Therefore, the algorithm leads to the important conclusion that even a highly efficacious AI, which can lead to disease stabilization, cannot prevent a tumor from regrowing upon cessation of therapy. This points to an inherent limitation in using AIs, even with cytotoxic chemotherapy, as a front-of-the-line cancer drug, at least for tumors growing in a vascular environment.

### 4.3. VDA in Isolation

Several vascular disrupting agents are currently undergoing testing in preclinical and clinical trials [4]. Although various mechanisms can be utilized to both target and disrupt the vasculature, the net effect of these drugs is to halt blood flow through the tumor vasculature and give rise to a widespread pattern of central necrosis [4]. In my simulations, I have used a generic VDA that shuts down the blood flow in the angiogenic vasculature, similar to the actions of the VDA CA4P.

As noted previously, VDAs are typically used in an intermittent fashion over shorter periods of time than an AI. For this reason, the VDA in the simulations was administered every three weeks, and because of the short half-life of CA4, the drugs action takes place only during the day the drug was administered. The effects of applying the VDA (when the vessel destruction parameter is *T*_3_ = 0.6) as described over a nine-month period of time can be seen in Figure 2. As with the other treatments, the VDA does limit tumor growth relative to applying no treatment whatsoever. However, the effects are not as promising as the other treatments tested. The area of the active tumor region continues to grow, albeit at a slower rate than when the VDA is not administered.

**Figure 7:**
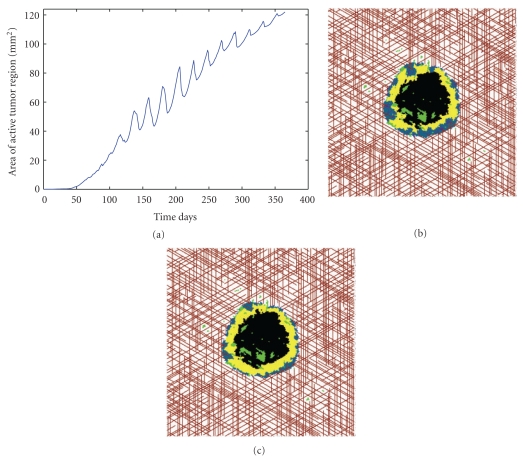
(a) Active area of a single tumor with *T*_3_ = 0.6. (b) Snapshot of tumor the day before VDA is administered. Notice the purple angiogenic vessels penetrating the tumor surface. (c) Snapshot of tumor the day after VDA is administered. Notice the absence of purple angiogenic vessels penetrating the tumor although red coopted vessels still supply the tumor with oxygen and nutrients.

In order to better understand tumor response to VDA treatment, in Figure 7(a) I have plotted the active tumor area as a function of time for a single tumor treated with a VDA (*T*_3_ = 0.6). This analysis shows that each day the VDA is administered, the active tumor area decreases for approximately one week. However, because this rapid vessel loss triggers massive amounts of hypoxia within the tumor, strong angiogenic signals are sent into the environment. The resulting angiogenesis causes the active tumor area to increase once again, and after only two weeks of applying the VDA, the active tumor area is typically restored to what it was before the previous treatment was applied. Therefore, given the time scales for angiogenesis in the model, I can conclude that administering a VDA once every three weeks is insufficient to maintain steady tumor growth inhibition.

The above observation explains the oscillatory behavior seen in the active tumor area plot (Figure 7(a)). However, it does not fully explain why the steady oscillations do not drastically limit active tumor size in the long-term. In order to fully understand the ineffectiveness of the VDA, it is useful to look at snapshots of the tumor directly preceding (Figure 7(b)) and proceeding (Figure 7(c)) VDA administration. Before VDA administration, the tumor is vascularized by both coopted (shown in red) and angiogenic (shown in purple) blood vessels. After applying the VDA, the majority of the angiogenic vessels are lost in the tumor mass. However, because VDAs work by selectively binding to angiogenic vessels, the coopted vessels in the tumor are not destroyed by VDA administration. Therefore, proliferative cancer cells that receive oxygen and nutrients via the coopted vasculature survive at the tumor periphery, and these cells maintain the growth of the tumor, just as seen in preclinical trials [4, 55]. Therefore, I have found that the combination of angiogenesis occurring between treatments, and slow growth occurring at the tumor periphery, greatly limits the antitumor activity of the simulated VDAs.

**Figure 8:**
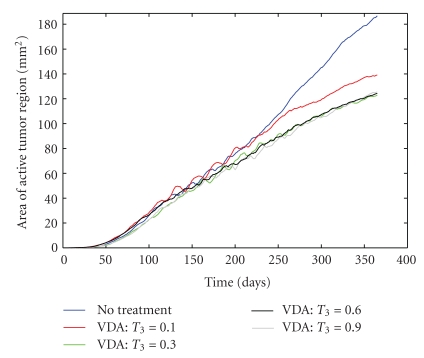
Sensitivity analysis of the VDA treatment parameter, *T*_3_. The average area of the active tumor region predicted by the algorithm is shown for each parameter value.

It is natural to ask whether the VDA being used in the simulations is not destroying enough angiogenic blood vessels (with *T*_3_ = 0.6), and if this is partially responsible for the low antitumor activity of the simulated VDA. Therefore, a sensitivity analysis was performed on *T*_3_, the VDA treatment parameter (Figure 8). Surprisingly, I find that increasing the VDA parameter beyond *T*_3_ = 0.3 has no measurable impact on the active tumor area. While this may seem improbable, it can be explained by understanding how vessel regression works in the algorithm. Each edge of the lattice that contains an angiogenic vessel is checked to see if it regresses. If regression occurs, not only does that “edge” of the vessel get destroyed, but any lattice edges that are upstream of that edge also get destroyed. In other words, if you kill the source of blood, any vessels that only received blood from that source are also effectively destroyed. This creates the relative insensitivity to changes in the VDA parameter *T*_3_. It is worth noting that if the VDA parameter is made sufficiently small (*T*_3_ = 0.1), the tumors grow at a noticeably faster rate than for *T*_3_ ≥ 0.3.

## 5. Conclusions

A previously validated mathematical model has been utilized to make predictions on the efficacy of certain vascular-targeting drugs. Three preclinically and/or clinically tested treatment protocols were analyzed, and simulation data was found to be in good agreement with the data collected from these trials. In particular, the antitumor activity of an angiogenesis inhibitor such as bevacizumab was well-predicted: there is an initial period of growth inhibition, but in the long-term, tumor regrowth occurs. The addition of a cytotoxic chemotherapeutic led to increased antitumor activity and was the most effective treatment tested. However, removing all drugs after one year of treatment restimulates tumor growth, suggesting that a protocol of an AI and a cytotoxic agent can increase progression free survival times, but cannot prevent long-term regrowth. The model also made predictions on the efficacy of a CA4P-like vascular disrupting agent that agreed with preclinical observations on the antitumor activity of VDAs. In particular, in spite of VDA administration, a rim of proliferative cells survive at the tumor periphery, and these, along with angiogenic activity in between drug administration, maintain steady tumor growth. The predictions made are highly dependent on the vascular nature of the tumor microenvironment, in which vessel cooption occurs alongside angiogenesis.

 Taken together, simulation and clinical data strongly suggest that vascular-targeting drugs, as currently administered, cannot lead to cancer eradication. However, long-term control may be possible, for instance, when a highly efficacious AI (*T*_1_ = 1000) is administered with a cytotoxic chemotherapeutic. While it certainly seems more desirable to eradicate an entire tumor mass, as compared to simply keeping the tumor at bay, recent work by Gatenby and colleagues suggests otherwise [56, 57]. In particular, taking lessons from both applied ecology and mathematical models, they suggest that therapeutic strategies that aim to maintain a stable, tolerable tumor volume have a better chance of success than those therapies that aim to maximize tumor killing. In this light, AIs may present themselves as a very important therapy. It still remains to be seen, however, whether this proposed paradigm shift will turn out to be a more successful way to treat cancer patients, or if maximizing cancer cell death is still the optimal way to proceed.

Any mathematical model has shortcomings that limit predictability, and some of the proposed model weaknesses warrant mentioning. One weakness is that there is only one pathway that leads to angiogenesis in the simulated tumors. In reality, there are many angiogenic pathways (although the VEGF pathway accounted for here is the dominant one) and treating a tumor with an AI that targets one pathway can cause a compensatory angiogenic response in the other pathways. In other words, unlike in my simulations, angiogenesis can occur in real tumors, even if the VEGF pathway is completely knocked down. Therefore, my simulations predict that less angiogenesis is occurring during AI administration than is likely the case. In spite of this shortcoming, the model is still able to identify an important feature of tumor growth in a vascular environment during AI treatment: the tumors can survive via the coopted vasculature, and slow growth is maintained. This suggests that drug cocktails that target multiple angiogenic pathways [5] may be able to slow tumor regrowth, but could not fully inhibit tumor expansion.

 Another shortcoming of the model is the nature in which blood is delivered to the tumor and how oxygen and drugs are distributed throughout the tumor mass. First of all, the model does not consider blood flow through the capillary network. This important modeling consideration has been taken up by other authors (see, e.g., [58–60]). Instead of modeling the details of blood flow, I have made the simplifying assumption that any cell within a fixed distance from a vessel receives an adequate supply of oxygen and drugs. This idealization ignores the fact that angiogenic blood vessels are leaky and may not homogeneously distribute oxygen and drugs throughout the tumor [1, 3]. Interestingly enough, despite the idealizations made, I have still been able to illustrate the failure of a number of treatment protocols. As future work, modifications can be made to improve the way in which drug transport and distribution are modeled. For instance, blood flow can be directly simulated or “pink noise” (i.e., a bandwidth-limited uncertainty) could be utilized to simulate the partially stochastic variations in vascular uptake. Another approach would be to couple a pharmacokinetic model of drug delivery and distribution with the hybrid cellular automaton model of tumor growth.

Finally, the model also assumes that there is a uniform phenotypic profile of cells within a tumor mass, which is never really the case. In future work, the model can be expanded upon to incorporate both interpatient and intertumor genotypic/phenotypic variability [41]. Further, not only is there phenotypic variability within a tumor, treatment can induce new mutations to occur in a cancer. In the future, the model will be expanded upon to incorporate the treatment-induced mutations.

To conclude, I have illustrated that the model can successfully predict, without any a priori knowledge, the antitumor activity of a number of vascular-targeting treatment protocols. The tumor microenvironment was shown to play an important role in drug activity. The predictions made by the model were verified by comparing to preclinical and clinical data wherever possible. The fact that the model could lead to predictions comparable to those made in preclinical and clinical trials is rather important. In order for clinical trials to reach these conclusions, millions of dollars were spent, many years of time were invested, and patients were put at risk of having an adverse response to the treatment. The work done herein illustrates that mathematical models can be used to test the efficacy of cancer drugs and, importantly, rule out drugs that will not have significant antitumor activity. In the future, I will use the mathematical model to test the efficacy of administering a drug cocktail of an AI and VDA, in an effort to learn if there are additive effects of combining these two vascular-targeting agents. I also plan to exploit the predictive abilities of the model to search for a treatment protocol that maximizes active tumor death, in the hopes of identifying a treatment that can cause permanent tumor growth inhibition.

## Figures and Tables

**Algorithm 1:**
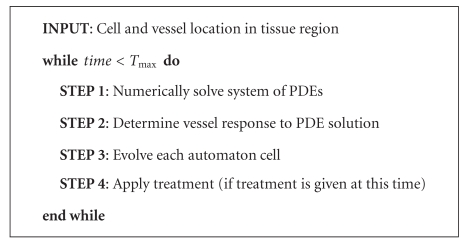
Hybrid CA model of vascular tumor growth and treatment.

**Table 1:** Summary of variables and parameters used in the model.

Variable	Definition	
*v*(*x*, *y*, *t*)	Concentration of VEGF (*μ*M)	
*a*_1_(*x*, *y*, *t*)	Concentration of Ang-1 (*μ*M)	
*a*_2_(*x*, *y*, *t*)	Concentration of Ang-2 (*μ*M)	
*r*_*v*0_(*x*, *y*, *t*)	Concentration of unbound VEGFR-2 (*μ*M)	
*r*_*a*0_(*x*, *y*, *t*)	Concentration of unbound Tie-2 (*μ*M)	
*r*_*v*_(*x*, *y*, *t*)	Concentration of VEGFR-2 bound by VEGF (*μ*M)	
*r*_*a*1_(*x*, *y*, *t*)	Concentration of Tie-2 bound by Ang-1 (*μ*M)	
*r*_*a*2_(*x*, *y*, *t*)	Concentration of Tie-2 bound by Ang-2 (*μ*M)	
*e*_*i*_(*x*, *y*, *t*)	EC indicator function	
*h*_*i*_(*x*, *y*, *t*)	Hypoxic cell indicator function	
*p*_*i*_(*x*, *y*, *t*)	Proliferative cell indicator function	
*n*_*i*_(*x*, *y*, *t*)	Necrotic cell indicator function	
*h*(*x*, *y*, *t*)	Concentration of hypoxic cells (*μ*M)	

PDE parameters	Definition	Value

*D*_*v*_	Diffusion coefficient of VEGF	*D*_*v*_ = 3.6 × 10^−4^ mm^2^/h
*D*_*a*2_	Diffusion coefficient of Ang-2	*D*_*a*2_ = 3.6 × 10^−4^ mm^2^/h
*b*_*v*_	Production rate of VEGF by hypoxic cells	*b*_*v*_ = 0.05 h^−1^
*b*_*a*1_	Production rate of Ang-1 by ECs	*b*_*a*1_ = 0.01 h^−1^
*b*_*a*2_	Production rate of Ang-2 by ECs	*b*_*a*2_ = 0.08 h^−1^
${\bar{b}}_{a2}$	Production rate of Ang-2 by hypoxic cells	${\bar{b}}_{a2}=0.05$ h^−1^
*μ*_*v*_	Decay rate of VEGF	*μ*_*v*_ = 0.001 h^−1^
*μ*_*a*1_	Decay rate of Ang-1	*μ*_*a*1_ = 0.003 h^−1^
*μ*_*a*2_	Decay rate of Ang-2	*μ*_*a*2_ = 0.002 h^−1^
*k*_0_	Association rate of VEGF/VEGFR-2	*k*_0_ = 46.8 *μ*M^−1^·h^−1^
*k*_−0_	Dissociation rate of VEGF/VEGFR-2	*k*_−0_ = 0.2268 h^−1^
*k*_1_	Association rate of Ang-1/Tie-2	*k*_1_ = 36 *μ*M^−1^·h^−1^
*k*_−1_	Dissociation rate of Ang-1/Tie-2	*k*_−1_ = 0.1332 h^−1^
*k*_2_	Association rate of Ang-2/Tie-2	*k*_2_ = 41.7 *μ*M^−1^·h^−1^
*k*_−2_	Dissociation rate of Ang-2/Tie-2	*k*_−2_ = 0.108 h^−1^
*K*_*v*_	Carrying capacity of VEGF	*K*_*v*_ = 10^−2^ *μ*M
*K*_*a*_	Carrying capacity of angiopoietins	*K*_*a*_ = 1.5 × 10^−2^ *μ*M
*e*_0_	Endothelial cell concentration per blood vessel	*e*_0_ = 10^−4^ *μ*M

Treatment parameters	Definition	Value

*T*_1_	AI treatment parameter is *b*_*v*_/*T*_1_	*T*_1_ = 10,100,1000
*T*_2_	Fraction of proliferative cells killed by cytotoxic agent	*T*_2_ = 0.24,0.34,0.44
*T*_3_	Fraction of angiogenic vessels destroyed by VDA	*T*_3_ = 0.1,0.3,0.6,0.9

**Table 2:** Dosing Schedule for Simulated Drugs.

Drug	Dosing Schedule	Therapeutic Levels?
AI	Once every two weeks	Maintained between successive treatments due to 20 day half-life of drug [43]

Cytotoxic Chemotherapeutic	Daily (up to 6 weeks in a row)	Maintained between successive treatments due to 1.8 hour half life of drug [44]

VDA	Once every three weeks	Maintained only in a 24 hour window after drug administration due to 4.2 hour half-life of drug [45]

## Appendix

### Estimating Cytotoxicity Parameter *T*_2_

The cell kill theory proposes that a set percent of proliferative cancer cells are killed with each dose of chemotherapy [51]. The log cell kill *L* is used to measure this percent

(A.1) $$\begin{array}{c} L={\log\;}_{10}\left(\frac{V_{\text{pre}}}{V_{\text{post}}}\right), \end{array}$$

where *V* is tumor volume, “pre” represents a pretreatment measurement, and “post” represents a posttreatment measurement. Assuming each cell has a fixed volume, it is straightforward to see that

(A.2) $$\begin{array}{c} L={\log\;}_{10}\left(\frac{N_{\text{pre}}}{N_{\text{post}}}\right), \end{array}$$

where *N* is the number of cells in the tumor. Solving for *N*_post_ gives

(A.3) $$\begin{array}{c} N_{\text{post}}=\frac{N_{\text{pre}}}{10^{L}}. \end{array}$$

In [51], mice were treated with temozolomide for five days. The log cell kill was calculated to be 0.4 log units. In order to use this information to determine the value of *T*_2_ (the percent of proliferative cells killed by temozolomide each day it is administered), I assume that the number of cells at time *t* is proportional to the number of cells at time *t* − 1, giving the discrete relationship

(A.4) $$\begin{array}{c} N\left(t\right)=k^{t}N_{\text{pre}}, \end{array}$$

where *k* is the growth proportionality constant. Using *L* = 0.4 and relationships (A.3) and (A.4), I find that after *t* = 5 days of treatment

(A.5) $$\begin{array}{c} N_{\text{post}}=N\left(5\right)=k^{5}N_{\text{pre}}=\frac{N_{\text{pre}}}{10^{0.4}}, \end{array}$$

which corresponds to *k* satisfying

(A.6) $$\begin{array}{c} k={\left(\frac{2}{5}\right)}^{1/5}\approx 0.83255. \end{array}$$

Therefore, the fraction of cells lost per day (looking at the net effect of growth and death) is 1 − *k* ≈ 0.16745, meaning the killing rate is *α* = −0.16745.

For GBM, the cell doubling time has been estimated to be approximately four days [31, 40]. Assuming exponential tumor growth, the growth rate of the tumor *r* can be calculated using the relation

(A.7) $$\begin{array}{c} N\left(t\right)=N_{\text{pre}}e^{rt}. \end{array}$$

Using the fact that the cell doubling time is approximately four days gives *r* ≈ 0.17563 as the expected growth rate. I can therefore represent the rate of cell lose per day as

(A.8) $$\begin{array}{c} \alpha =r-T_{2}\Leftrightarrow \text{net}\;\text{growth}=\text{rate}\;\text{of}\;\text{growth}-\text{killing}\;\text{rate}. \end{array}$$

This implies that *T*_2_ = 0.343, and, therefore, I use *T*_2_ = 0.34 as the baseline value for the cytotoxicity of temozolomide.
